# Programs of religious/spiritual support in hospitals - five “Whies” and five “Hows”

**DOI:** 10.1186/s13010-016-0039-z

**Published:** 2016-08-22

**Authors:** Marcelo Saad, Roberta de Medeiros

**Affiliations:** 1 Universidade de Santo Amaro, Rua Montesquieu 371, CEP 04116-190 São Paulo, SP Brazil; 2 Centro Universitário São Camilo, Rua Montesquieu 371, CEP 04116-190 São Paulo, SP Brazil

**Keywords:** Pastoral care, Chaplaincy hospital service, Religion and medicine, Spirituality, Humanism

## Abstract

A contemporary orientation of the hospital experience model must encompass the clients’ religious-spiritual dimension. The objective of this paper is to share a previous experience, highlighting at least five reasons hospitals should invest in this direction, and an equal number of steps required to achieve it. In the first part, the text discourses about five reasons to invest in religious-spiritual support programs: 1. Religious-spiritual wellbeing is related to better health; 2. Religious-spiritual appreciation is a standard for hospital accreditation; 3. To undo religious-spiritual misunderstandings that can affect treatment; 4. Patients demand a religious-spiritual outlook from the institution; and 5. Costs may be reduced with religious-spiritual support. In the second part, the text suggests five steps to implement religious-spiritual support programs: 1. Deep institutional involvement; 2. Formal staff training; 3. Infrastructure and resources; 4. Adjustment of institutional politics; and 5. Agreement with religious-spiritual leaders. The authors hope the information compiled here can inspire hospitals to adopt actions toward optimization of the healing experience.

## Background

*‘There is no profit in curing the body if in the process we destroy the soul’ (Samuel H. Golter, author of “The City of Hope”, 1890–1971).*

A person’s confrontation with serious illness or injury and the looming possibility of death raises primary spiritual questions. Notedly, the inpatient is more vulnerable, since he/she is away from his/her pillars of faith, such as community resources and daily rituals. The hospital should provide religious/spiritual resources in order to promote the most effective coping strategy to their patients, considering either recovery or possible death [1]. Spirituality may be defined as the search for ultimate meaning, purpose and significance, in relation to oneself, family, others, community, nature and “sacred”, expressed through beliefs, values, traditions and practices [2]. Many people express their spirituality trough their formal religions or their traditional faiths. Others still strengthen their spiritual dimension with non-religious elements. Although religiosity and spirituality are distinct constructs, the overlap between them is remarkable and consistent. Thus, the term religiosity/spirituality (R/S) is often adopted to refer to transcendent elements of meaning, purpose and connectedness. A contemporary orientation of the hospital experience model, therefore, must encompass the spiritual dimension.

The main author of the present paper acquired experience in this development between 2009 and 2011, when he provide advice to the Spirituality Committee of the *Hospital Israelita Albert Einstein*, in S. Paulo, Brazil (http://apps.einstein.br/english). This committee, along with other ones, was part of the preparation for the Planetree accreditation (http://planetree.org) The Planetree certifies health institutions who value the patients well-being and their families by providing healthy environments, propitious for healing, providing a humane treatment experience. Spirituality is one of the 10 pillars of the Planetree philosophy, in which it is emphasized hospitals should support the spiritual needs of their patients. In December 2011, the institution, one of the most appreciated hospitals in Latin America, became the first one to obtain such accreditation in this subcontinent. As a result, the hospital became the regional responsible for training other health institutions interested in the Planetree model.

The objective of this paper is to share this experience, highlighting at least five reasons hospitals should invest in this direction, and an equal number of steps required to achieve it. This paper does not have the ambition to exhaust the matter, but instead to be a starting point to discuss the complex issue of how to meet R/S needs concurrently with adequate health treatment for inpatients. Besides the historical religions, there are many legally constituted beliefs and informal faith traditions, each one with particular ethical values and moral recommendations. Thus, to select a unique way to offer support the inpatients on their special needs for the sustainment of their faith is a challenge. The importance of R/S support is greater in communities where religion represents a social norm (i.e., it is common and socially desirable). The advancement of initiatives on spiritual support in hospitals depends also on the demand of the regional characteristics of populations. Therefore, the authors recommend a careful assessment of the local reality to all those who plan to deploy a project of this kind.

## Five reasons to invest in religious/spiritual support programs

### Religious/spiritual wellbeing is related to better health

Multiple well-designed studies (with control for confounding factors) have demonstrated patients who attend religious services, independently of denomination, have better long-term health-care outcomes. Religiosity and spiritual experiences are especially associated with better physical and mental health parameters; less need for health services; faster recovery from illness; and increased longevity [3]. Instead, religious/spiritual coping may sometimes be negative when it is based on guilt or resentment, creating religious/spiritual suffering that must be identified and addressed, otherwise affecting adversely the course of disease, despite the best medical treatment.

### Religious/spiritual appreciation is a standard for hospital accreditation

Joint Commission International (JCI) demands, on Patient and Family Rights section [4]: “*The hospital provides care that is respectful of the patient’s personal values and beliefs and responds to requests related to spiritual and religious beliefs*”. JCI do not specify what needs to be included to accomplish such demand. Each hospital is free to define the content and scope of religious/spiritual assessment and the qualifications of the individuals performing it, but JCI suggests forms to identify and accommodate patient cultural, religious or spiritual beliefs and practices that influence care [5]. In a routine screening, desirable items include the record of the belief system (affiliation); level of religious observance; involvement with religious community; and rituals particularly importante.

### To undo religious/spiritual misunderstandings that can affect treatment

Faith systems (either from religious doctrines or from cultural traditions) provide moral guidance about a variety of health issues and prescribe or proscribe many behaviors, which can have medically related consequences. Sometimes a belief outlook of patient or family can affect clinical decisions in issues such as circumcision, blood transfusion, life-sustaining suspension, organ donation and necropsy. Not all patients fully understand the beliefs of their own denominations about particular ethical issues, and so pastoral care staff or the patient’s own clergy can sometimes be of enormous help in clarifying for patients what their own traditions hold to be true [6]. The presence of the clergyman may be especially important when the patient has one religious affiliation and the relatives have another, as it happens frequently in the current multicultural societies. An intermediation is also welcome when the religious perspectives from healthcare professionals affect clinical decisions, especially on sensitive issues such as termination of pregnancy or treatment suspension in terminal illnesses.

### Patients demand a religious/spiritual outlook from the institution

Several studies show the majority of patients want their physicians to acknowledge religious/spiritual issues and address their spiritual needs [7]. Many patients expect and appreciate it if the physician consider his/her spiritual needs, especially in life-threatening circumstances, serious medical conditions and loss of loved ones [8]. Attention to patients’ spiritual needs can improve the quality of communication among clinicians, patients and families and reduce the gap between the health care patients want and expect and what they receive [9]. Meeting spiritual needs is correlated with patient satisfaction with care and patient ratings of the quality of medical care [10]. Chaplain visit may increase patient satisfaction after discharge, and it may be associated with significantly greater overall satisfaction with care given, better rating of hospital stay, and greater likelihood of recommending the hospital [11].

### Costs may be reduced with religious/spiritual support

A study [12] among advanced cancer patients near death showed those with poorly supported religious/spiritual needs had significantly higher costs, more intensive care unit stays, and less hospice stays. In the opposite way, patients near death whose spiritual needs were supported scored higher on quality of life and were significantly more likely to use hospice care, and those patients with high religious/spiritual coping were also less likely to receive aggressive end-of-life care [13, 14]. Whether the spiritual support could reduce or not the hospital lenght of stay is still under discussion. However, hospital discharge may be faster if the patient is well connected to his/her religious/spiritual community, where there is a network that can provide emotional support, monitor treatment compliance and provide services such as meals and rides to physician office.

## Five steps to implement religious/spiritual support programs

### Deep institutional involvement

An important action is to link the religious/spiritual support program to a major humanistic project of the institution, in order to guarantee the resources for its continuation, among many other financial priorities. The hospital must truly embrace the idea, in order to provide assistance that consider and respect the values of the patient. The institution must also aknowledge all the customers of spiritual support, encompassing not only the patients, but including the family, the caregivers and the healthcare staff. This last group is frequently forgot, although “to take care of who takes care” is essencial.

### Formal staff training

As a rule, healthcare professionals do not receive at graduation straight training about religious/spiritual issues related to the clinical routine. To prepare healthcare professionals to understand and protect religious/spiritual values of clientes, a formal training should be provided to all hospital staff. Even those professionals not directly in contact with patients must ideally participate. Training can be done by a distant learning resource, such as a short class recorded as an electronical movie file, available on the intranet of the institution. The content may include the importance of relations between personal religiousness/spirituality and health and disease, and the implications of such relations over the treatment and healing processes. It would also be useful to list some attitudes that the healthcare professional should perform and those that should be avoided.

### Infrastructure and resources

The institution must clearly declare its commitment to support the clients’ religious/spiritual needs. Institution must inform patients at hospital admission about their rights and the resources available there. This statement may be a clear paragraph in the institutional brochure provided to the patient on admission. A large numbers of patients report a wide spectrum of spiritual needs, such as help with specific religious/spiritual rituals. A basic infrastructure to support these needs include [15]: a private space for contemplation and reflection (many times the hospital chapel); diversified medias (sacred books, inspiring DVDs, etc.); some ritualistic equipment (including adaptations such as electronic candles). Support groups, either religion-based or not, are useful for individual, family or small groups interventions.

### Adjustment of institutional politics

A whole set of pastoral interventions includes [16]: assessment (welfare, needs and resources of the patient); ministry (relationship, conversation and company support); counseling (ethics consultation and education); and ritual (support on religious practices, such as the Eucharist or other sacraments). Many conflicts with hospital routine may surge in such sequency, such as lack of privacy, procedure disturbance, lack of time and inadequate moments for visiting. Sometimes, hospital rules such as visiting hours or number of visitors must be relaxed. Little concessions must be done for the patient whenever possible, from the preference to be attended by a professional of the same gender, until to consult a religious/spiritual leader before accepting a procedure. In order not to hurt sensibilities or be invasive, the ideal situation is to check with the patient, on admission, whether he/she want the religious/spiritual visit. If so, the patient name goes into a list that is provided to the clergyman, who then makes the religious visit only for them, avoiding an inopportune intrusion.

### Agreement with religious/spiritual leaders

In some cases, the patient’s own cleric is the one who provide the spiritual care, if this cleric is able to come to the hospital. However, pastoral care for the inpatient is the special domain of certified healthcare chaplains. About two-thirds of U.S. hospitals have such professionals [17], although it is not the reality in many countries. If the hospital has not a chaplain, external clergymen may do occasional visits (upon request) or regular ones (in a partnership between the hospital and a congregation). In healthcare institutions, chaplains typically strive to serve people of many different denominations, in a multifaith effort. Nevertheless, some patients may have a syncretistic set of beliefs from different traditions, or they may find transcendence in art or in a humanistic phylosophy. For such eclectic needs, laic volunteers may work to support universal needs without discussing beliefs, with presence, compassion and understanding.

## Conclusion

*‘Patients and physicians have begun to realize the value of elements such as faith, hope and compassion in the healing process. The value of such spiritual elements in health and quality of life has led to more holistic view of health that includes a non-material dimension’.* This affirmation from the World Health Organization [18] exemplifies the progressive acceptance of religiousness/spirituality as an important aspect of the path for cure. The interface between religion/spirituality and health can be provocative and controversial, but it must be included in the hospital therapeutic context. To minimize the importance of this dimension would be a failure to implement a full patient-centered care. The authors hope the information compiled here can inspire hospitals to adopt actions toward optimization of the healing experience.

## Abbreviations

JCI: Joint Commission International

## References

[1] VandeCreek L (2010). Defining and advocating for spiritual care in the hospital. J Pastoral Care Counsel.

[2] Saad M, de Medeiros R, Saad M, de Medeiros R (2012). Religious/spiritual Coping – Health Services Empowering Patients’ Resources. Complementary therapies for the contemporary healthcare.

[3] Koenig HG (2012). Religion, spirituality, and health: the research and clinical implications. ISRN Psychiatry.

[4] Joint Commission International Accreditation Standards For Hospitals. 5th. Ed. 2014. Available from http://www.jointcommissioninternational.org/assets/3/7/Hospital-5E-Standards-Only-Mar2014.pdf. Accessed 4 Jan 2016.

[5] The Joint Commission. Advancing Effective communication, cultural competence, and patient- and family-centered care: a roadmap for hospitals. Oakbrook Terrace: The Joint Commission; 2010. Available from http://www.jointcommission.org/assets/1/6/ARoadmapforHospitalsfinalversion727.pdf. Accessed 4 Jan 2016

[6] Sulmasy DP (2009). Spirituality, religion, and clinical care. Chest.

[7] Chattopadhyay S (2007). Religion, spirituality, health and medicine: Why should Indian physicians care?. J Postgrad Med.

[8] McCord G, Gilchrist VJ, Grossman SD, King BD, McCormick KE, Oprandi AM (2004). Discussing spirituality with patients: a rational and ethical approach. Ann Fam Med.

[9] Edwards A, Pang N, Shiu V, Chan C (2010). The understanding of spirituality and the potential role of spiritual care in end-of-life and palliative care: A meta-study of qualitative research. Palliat Med.

[10] Sulmasy DP (2007). Distinguishing denial from authentic faith in miracles: a clinical-pastoral approach. South Med J.

[11] Marin DB, Sharma V, Sosunov E, Egorova N, Goldstein R, Handzo GF (2015). Relationship between chaplain visits and patient satisfaction. J Health Care Chaplain.

[12] Balboni T, Balboni M, Paulk ME, Phelps A, Wright A, Peteet J (2011). Support of cancer patients’ spiritual needs and associations with medical care costs at the end of life. Cancer.

[13] Balboni TA, Paulk ME, Balboni MJ, Phelps AC, Loggers ET, Wright AA (2010). Provision of spiritual care to patients with advanced cancer: associations with medical care and quality of life near death. J Clin Oncol.

[14] Flannelly KJ, Emanuel LL, Handzo GF, Galek K, Silton NR, Carlson M (2012). A national study of chaplaincy services and end-of-life outcomes. BMC Palliat Care.

[15] Clark PA, Drain M, Malone MP (2003). Addressing patients’ emotional and spiritual needs. Jt Comm J Qual Saf.

[16] World Health Organization (2002). Pastoral Intervention Codings, International Classification of Diseases, ICD-10-AM.

[17] Committee on Approaching Death; Institute of Medicine. Dying in America. Institute of Medicine of the National Academies. Washington: The National Academies Press; 2014. ISBN 978-0-309-30310-1. Available at http://www.ncbi.nlm.nih.gov/books/NBK285681/. Accessed 4 Jan 2016.

[18] WHOQOL SRPB Group. WHOQOL and spirituality, religiousness and personal beliefs: report on WHO consultation. World Health Organization, 1998. WHO/MSA/MHP/98.2, 2–23. Geneva, Switzerland

